# TNF inhibitors increase the risk of nontuberculous mycobacteria in patients with seropositive rheumatoid arthritis in a mycobacterium tuberculosis endemic area

**DOI:** 10.1038/s41598-022-07968-w

**Published:** 2022-03-07

**Authors:** Dong Won Park, Yun Jin Kim, Yoon-Kyoung Sung, Sung Jun Chung, Yoomi Yeo, Tai Sun Park, Hyun Lee, Ji-Yong Moon, Sang-Heon Kim, Tae-Hyung Kim, Ho Joo Yoon, Jang Won Sohn

**Affiliations:** 1 Department of Internal Medicine, Hanyang University College of Medicine, Seoul, Republic of Korea; 2 Biostatistical Consulting and Research Lab, Medical Research Collaborating Center, Hanyang University, Seoul, Republic of Korea; 3 Department of Rheumatology, Hanyang University Hospital for Rheumatic Diseases, Seoul, Republic of Korea; 4 Present Address: Department of Internal Medicine, Hanyang University College of Medicine, 222-1 Wangsimni-ro, Seongdong-gu, Seoul, 04763 Republic of Korea

**Keywords:** Infectious-disease epidemiology, Rheumatic diseases

## Abstract

The aim of this study is to examine the impact of tumor necrosis factor inhibitors (TNFI) on nontuberculous mycobacterium (NTM) infection in rheumatoid arthritis (RA) patients in a mycobacterium tuberculosis (MTB) endemic area. We selected 1089 TNFI-treated RA patients and 4356 untreated RA patients using propensity-matching analysis according to age, gender, and Charlson comorbidity index using the Korean National Health Insurance Service database from July 2009 to December 2010. Both groups were followed-up until the end of 2016 to measure the incidence of mycobacterial diseases. The incidence rate of NTM in TNFI-treated RA group was similar to those of MTB (328.1 and 340.9 per 100,000 person-years, respectively). The adjusted hazard ratio (aHR) of NTM for TNFI-treated RA compared to untreated RA was 1.751(95% CI 1.105–2.774). The risk of TNFI-associated NTM in RA was 2.108-fold higher among women than men. The age-stratified effects of TNFI on NTM development were significantly high in RA patients aged 50–65 years (aHR 2.018). RA patients without comorbidities had a higher incidence of NTM following TNFI treatment (aHR 1.742). This real-world, observational study highlights the need to increase awareness of NTM in TNFI-treated RA patients in an MTB endemic area.

## Introduction

Rheumatoid arthritis (RA) is a chronic inflammatory disorder that increases susceptibility to infection^1^. The advent of biological disease-modifying anti-rheumatic drugs (DMARDs), including tumor necrosis factor inhibitors (TNFIs), has revolutionized RA treatment, reducing joint damage and giving better clinical outcomes^2,3^. These drugs reduce the action of TNF-α, a pro-inflammatory cytokine that causes synovitis in RA. However, TNFIs also play an important role in the immune response to mycobacteria^4^, and can increase the occurrence of opportunistic infections, particularly by mycobacterium tuberculosis (MTB)^5,6^. The increased risk of MTB development in RA patients treated with TNFI is currently recognized, which is the rationale behind screening for and treating LTBI prior to TNFI^7^.

Recently, nontuberculous mycobacteria (NTM) disease has emerged as another mycobacterial infection in patients receiving TNFI globally^8^. Although NTM are not typically transmissible, they can result in severe, predominantly pulmonary infections^9^. However, little is known about the association between TNFI, RA and NTM disease, although some increased risk is clearly suggested. NTM may act as opportunistic pathogens, exploiting impaired airway epithelium^10^ or immunocompromised condition^9^, and so may pose a risk to RA patients. The literature currently suggests that RA patients are at higher risk of developing NTM than the general population^11,12^, with TNFI being an important additional predisposing factor^13,14^. To date, previous studies on NTM infection been conducted in regions where NTM infection is more common than MTB infection, such as the US and Canada. Few studies have examined the important issue of whether RA patients receiving TNFI are at increased risk of NTM in TB endemic areas^15^.

This study determines the incidence rates of NTM and MTB considering TNFI use, and investigates whether TNFI increases the risk of NTM in RA patients in the MTB endemic area. We followed a population-based, longitudinal cohort of seropositive RA patients receiving TNFI using data from the Korean National Health Insurance Service (NHIS) and compared this cohort with a 1:4 propensity-matched cohort of untreated RA patients in an intermediate TB burden country, South Korea.

## Results

### Characteristics of the study population

**Figure 1:**
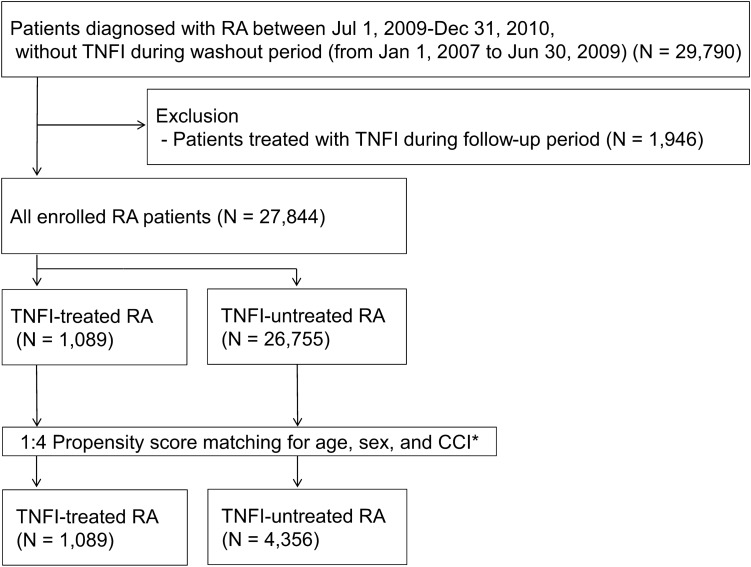
Flow chart of the study population. CCI, Charlson comorbidity index; RA, rheumatoid arthritis; TB, tuberculosis; TNFI, tumor necrosis factor inhibitor.

The baseline cohort comprised 27,844 RA patients, of whom 1089 (3.91%) were TNFI-treated and 26,755 were untreated (Fig. 1). After propensity score matching, all selected patients formed well-matched 1:4 pairs in RA patients. The study cohort included 1089 TNFI-treated RA patients matched with 4356 untreated RA patients, and the mean follow-up duration were 89.7 ± 7.6 and 84.4 ± 7.9 months, respectively. As shown in Table 1, the baseline characteristics were well balanced between the two groups with regard to age, gender, and CCI scores.

**Table 1:** Baseline characteristics of all enrolled rheumatoid arthritis (RA) patients after propensity score matching.

Variable	All RA (N = 5445)		TNFI untreated RA group (N = 4356)		TNFI treated RA group (N = 1089)		*p-*value
**Gender**							
Male	883	16.22	703	16.14	180	16.53	0.7547
Female	4562	83.78	3653	83.86	909	83.47	
**Age**							
Mean ± SD	52.62 ± 12.89		52.62 ± 12.89		52.62 ± 12.89		1.0000
18 ≤ age < 50	2040	37.47	1632	37.47	408	37.47	1.0000
50 ≤ age < 65	2365	43.43	1892	43.43	473	43.43	
65 ≤ age	1040	19.10	832	19.10	208	19.10	
**CCI**							
Mean ± SD	1.23 ± 0.57		1.23 ± 0.56		1.25 ± 0.6		0.7417
0	4203	77.19	3378	77.55	825	75.76	0.3483
1–2	1189	21..84	939	21.56	250	22.96	
≥ 3	53	0.97	39	0.90	14	1.29	

*CCI* Charlson comorbidity index, *RA* rheumatoid arthritis, *SD* standard deviations, *TB* tuberculosis, *TNFI* tumor necrosis factor inhibitor.

### The risk of developing NTM in RA with and without TNFI treatment

**Figure 2:**
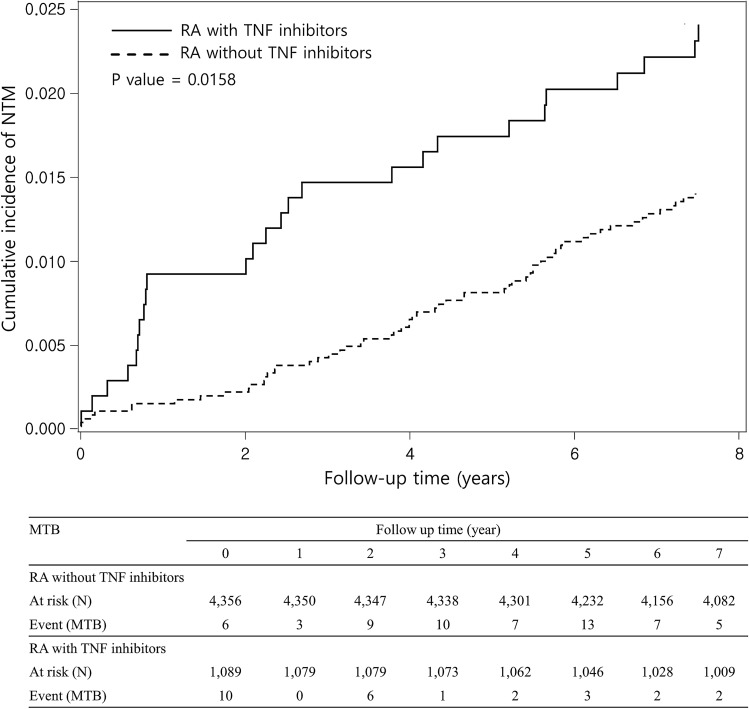
Cumulative incidence of NTM for TNFI-treated RA (solid line) and untreated RA (dashed line) for matched cohorts. NTM, nontuberculosis mycobacterium; RA, rheumatoid arthritis; TNFI, tumor necrosis factor inhibitor.

Among 5445 RA patients, 86 (1.58%) and 107 (1.97%) were found to be have NTM and MTB during the 86.0 months (median) follow-up, respectively. A total of 26 NTM and 27 MTB cases occurred after starting TNFI treatment in the 1089 TNFI-treated RA patients. The median duration from initiation of TNFI therapy to development of NTM and MTB was 29.4 and 16.4 months, respectively. The incidence rate of NTM disease in TNFI-treated RA group was similar to those of MTB disease (328.1 and 340.9 per 100,000 person-years, PY, respectively). The incidence of NTM disease was 1.750 times greater in TNFI-treated RA than untreated RA (328.1 vs. 187.4 per 100,000 PY, respectively), with an adjusted HR (aHR) of 1.751 (95% CI 1.105–2.774). The risk of developing NTM after TNFI treatment was significantly greater for female RA patients than male patients (aHR 2.108, 95% CI 1.287–3.453). The risk of NTM after TNFI treatment was also significantly increased in RA patients in the age ranges of 50–65 years (aHR 2.018, 95% CI 1.062–3.833). Patients without comorbidity had a higher aHR of 1.742 (95% CI 1.019–2.978) for NTM disease in TNFI-treated RA than in untreated RA (Table 2). The incidence of MTB was 1.358 times greater in TNFI-treated RA than untreated RA (340.9 vs. 251.1 per 100,000 PY, aHR 1.352, 95% CI 0.874–2.091), but this difference was not significant (Table 2). As shown in Fig. 2, Kaplan–Meier analysis revealed that the cumulative incidence of NTM was significantly higher in TNFI-treated RA than untreated RA (*p* = 0.0158).

**Table 2:** Risks of NTM and MTB measured by sex, age, and Charlson index score for matched cohorts.

Variable	TNFI untreated RA group (N = 4356/ 31,863 PY)			TNFI treated RA group (N = 1089/ 7920 PY)			Compared to TNFI untreated RA group	
	Event	PY	Rate^#^	Event	PY	Rate^#^	IRR* (95% CI)	Adjusted HR^†^ (95% CI)
MTB	80	31,863	251.1	27	7920	340.9	1.358 (0.844–2.124)	1.352 (0.874–2.091)
NTM	60	32,011	187.4	26	7925	328.1	1.750 (1.060–2.817)**	1.751 (1.105–2.774)**
**Gender**								
Male	14	5089	275.1	2	1290	155.0	0.563 (0.062–2.453)	0.570 (0.129–2.510)
Female	46	26,921	170.9	24	6635	361.7	2.117 (1.236–3.541)**	2.108 (1.287–3.453)**
**Age**								
18 ≤ age < 50	12	12,199	9.84	6	3039	19.74	2.007 (0.618–5.776)	1.997 (0.749–5.326)
50 ≤ age < 65	28	13,997	20.00	14	3451	40.57	2.028 (0.987–3.983)	2.018 (1.062–3.833)**
65 ≤ age	20	5816	34.39	6	1435	41.82	1.216 (0.400–3.139)	1.199 (0.481–2.987)
**CCI**								
0	45	24,837	181.2	19	5999	316.7	1.748 (0.966–3.05)	1.742 (1.019–2.978)**
≥ 1	15	7174	209.1	7	1926	363.4	1.738 (0.600–4.529)	1.766 (0.720–4.334)

Rate^#^, incidence rate, per 100,000 person-years; IRR*, incidence rate ratio; Adjusted HR^†^; model manual adjusted for sex, age, and CCI score; **p < 0.001.

*CCI* Charlson comorbidity index, *CI* confidence interval, *HR* hazard ratio, *MTB* mycobacterium tuberculosis, *NTM* nontuberculosis mycobacterium, *PY* person-year, *RA* rheumatoid arthritis, *TB* tuberculosis, *TNFI* tumor necrosis factor inhibitor.

### Discussion

Using a large-scale, population-based, longitudinal cohort study, we demonstrated that TNFI treatment increases the risk of developing NTM in seropositive RA patients (aHR 1.751) in the TB endemic area of South Korea. The risk of TNFI-associated NTM development in RA was 2.108-fold higher among women than men. The age-stratified effects of TNFI on NTM development were significantly high in RA patients aged 50–65 years (aHR 2.018). RA patients without comorbidities had a higher incidence of NTM disease following TNFI treatment (aHR 1.742).

NTM and MTB disease have similar symptoms and radiographic characteristics, making it difficult to distinguish between them. Physicians are more likely to focus on MTB disease because of its relatively high incidence in MTB endemic areas, including South Korea^16^. The epidemiological relationship between NTM and MTB could be partly explained by this clinical confusion^17^. In western countries, the incidence rate of MTB is relatively low and the burden of NTM far outstrips that of MTB^18^. This trend seems to hold true in the impact of TNFI on development of mycobacterial infection in RA patients. Previous studies in the US reported that NTM cases are nearly twice as frequent as MTB cases in patients receiving TNFI^14,19^. However, we showed NTM incidence (328.1 per 100,000 PY) was similar to those of MTB (340.9 per 100,000 PY), which is not surprising considered that our study was conducted in MTB endemic area. Moreover, another US study showed that the incidence of NTM was significantly higher in TNFI-treated RA than untreated RA^14^. A case–control study also reported that senior Canadians with NTM and MTB were more likely to be under current TNFI (adjusted ORs 5.04, and 2.19, respectively) compared with non-TNFI users^13^. On the other hand, a case–control study in an MTB endemic area by Liao et al. found that although TNFI-treated RA patients had an increased risk of NTM infections compared to untreated RA patients, there was no statistical significance after adjusting for variables^15^. This decreased statistical power might be related to small case numbers. Extending those findings with a large population-based longitudinal RA cohort study, we showed that NTM is significantly more common in TNFI-exposed RA patients than unexposed patients. These findings suggest that TNFI poses a risk to the occurrence of NTM regardless of the prevalence of MTB.

The risk of NTM increases with age, both worldwide^20^, and in South Korea^21^, as failure to control infection is more likely in elderly patients with comorbidities. Previous studies indicated that advanced age (≥ 65 years) was a significant risk factor for the development of NTM diseases in the RA cohort^22^, even in patients receiving TNFI^14^. However, this study showed that the risk of NTM disease significantly increased in RA patients aged 50–65 years. Interestingly, the impact of TNFI on NTM development was found to be significantly high in middle-aged RA patients as well as in elderly RA patients. Patient-specific risk factors for developing NTM may vary with geographical area and disease characteristics. RA may be associated with increased risk of all forms of NTM, including pulmonary and extrapulmonary forms^12^. By reviewing the MedWatch database for NTM cases in the US, Winthrop et al. reported that 44% of extrapulmonary NTM occurred in patients receiving TNFI^23^. In western countries, disseminated NTM diseases seem to occur at a considerable rate in RA patients with TNFI^12,14^. However, in South Korea, pulmonary NTM diseases seem to predominate over extrapulmonary cases in RA patients. Lim et al. evaluated NTM prevalence in South Korean RA patients and reported no extrapulmonary involvement in any of the cases^24^. Another South Korean study on the clinical characteristics of NTM disease during TNFI therapy reported only six NTM patients with pulmonary complications^8^. Considering these results, our study seems to reveal a relatively large proportion of pulmonary NTM diseases, although, due to study methodologies, it was not possible to determine the involvement of NTM. NTM lung disease almost exclusively occurs in individuals over 45 years old with underlying lung disease, such as bronchiectasis and interstitial lung disease^25^. Recently, bronchiectasis has been observed in up to 30% of RA patients^26^, and such modifications of the pulmonary structure may provide a favorable environment for infection and colonization with NTM organisms^27^. A previous population-based study showed that younger bronchiectasis patients had a higher risk of NTM growth^28^. This may have affected the risk of TNFI-induced NTM in our RA cohort. Further research is required to explore the association of NTM with young and middle aged TNFI-exposed RA patients.

We found that the RA cohort without CCI displayed a 1.742-fold higher risk of developing NTM disease than the comparison cohort, supporting the theory that TNFI could play a critical role for development of NTM disease^11^. Our study also showed that the TNFI-associated risk of NTM disease in RA was significantly increased in females. Reports of gender differences in the incidence of NTM vary in the literature. Previous reports have shown that women predominated the NTM cases in RA patients receiving TNFI^8,23^. However, other studies have shown no difference in the development of TNFI-associated NTM between men and women^14^. One study in Taiwan reported that the risk of NTM disease was even higher among male RA patients^22^. More studies are therefore needed to address the impact of gender on NTM development in TNFI-treated RA patients.

The TNFI treatment could increase the risk of progression of both latent and newly acquired MTB infection by restricting macrophage function to control the growth of intracellular MTB^29^. However, regarding NTM, for which latent infection is not recognized, harmful effects of TNFI are likely limited to promoting the acquisition of new NTM infection or the progression of an existing infection^30^. The difference in the TNFI related risks between MTB and NTM provide clinical implications that strategies to prevent MTB and NTM should be different. Screening and treating LTBI prior to use of TNFI have clear evidence to reduce the risk of MTB infection^7^. Current evidence might not yet be sufficient to recommend screening and prevention for NTM infection prior to starting TNFI in RA patients. We demonstrated TNFI treatment in RA patients could be a risk factor for NTM disease, which may promote the progression of preexisting unrecognized NTM infection. NTM screening including chest imaging and systemic symptom review might result in early detection of NTM disease that require antimicrobial therapy in TNFI-treated RA patients.

This study was intended to focus on the impact of TNFI on the burden of incident NTM infection in RA patients. Regarding study design, however, several issues should be considered when interpreting our results. Although we implemented a relatively long washout period of > 2 years to exclude cases previously treated TNFI, enrolled subjects could have taken TNFI before the washout period or in clinical trials. We could not exclude patients who had received diagnoses of NTM and MTB before washout period (2007–2009) because we only have data for 2007–2016. Our study applied the different definition of index date between TNFI-treated RA group and untreated RA group. TNFI treated RA group had a slightly longer follow-up period compared to untreated RA group (89.7 ± 7.6 and 84.4 ± 7.9 months, respectively), which may affect the incidence of NTM infection.

Our study had several limitations. First, the NHIS database does not provide detailed individual patient information, such as data on diagnosis year of RA or rheumatic disease activity, which has been associated with anti-rheumatic medication and opportunistic infection^31^. The American College of Rheumatology guidelines request that clinicians consider various factors, including comorbid conditions, disease activity, and previous use of DMARDs and other immunosuppressive agents, before prescribing TNFI to RA patients^32^. In clinical practice, TNFIs have restricted applications in patients with comorbid conditions such as heart failure, malignancy, or infection, including mycobacterial infections, which may affect the decision to prescribe TNFI for RA. Additionally, patients with more severe RA may be more likely to use biologics. Tiippana-Kinnunen et al. observed that comorbidities increased during the 15 years of RA and there was significant relationship between comorbidities and disease activity in both early RA disease and at end-point^33^. The CCI score might be related to the risk of mycobacterial infection in the RA cohort^11^.Various comorbidities in RA patients may also influence the discontinuation of TNFI^34^. High baseline CCI in RA patients also correlated with higher disease activity both in early RA disease and at end-point^33^. Considering these findings, we controlled for CCI, as well as age and gender, using propensity score matching. Second, since drug data such as data regarding treatment with non-TNF biologics, DMARDs, and corticosteroids were not collected in our study, they were not considered in determining the outcomes in this study, which could be another limitation. As potent immunosuppressive drugs, corticosteroids therapy may increase the risk of NTM disease in RA patients in a dose-dependent manner^15^. Exposure to leflunomide and other anti-rheumatic drugs with high immunosuppressive potential also was associated with both TB and NTM disease in older RA patients^13^. Further investigation exploring the impact of TNFI on the incidence of NTM disease considering the administered drugs other than TNFI, which could shed more light on our speculation, will be needed. Third, we identified RA cases using RA diagnosis code from the ICD10 and the rare intractable disease (RID) database. This study may therefore include more severe RA patients restricted to those who visited secondary or tertiary medical institutions and required treatment in the RID database. Moreover, our study focuses only on seropositive RA patients identified using the RID database, which includes seropositive, but not seronegative RA. About 85% of RA patients in Korea are expected to have rheumatoid factor and anti-CCP antibody^35^. Thus, our data may not be generalizable to seronegative RA. Fourth, we defined NTM infection using only ICD-10 codes assigned by health care providers. NTM infection was mainly confirmed by NTM isolation from a respiratory source. This coding-based analysis may not include laboratory results, radiological findings, or bacteriologic results, and instead relies on physician judgment for diagnosis. Recent NTM epidemiological studies in South Korea have demonstrated the reliability of using this method to define NTM disease^21,36^. Despite, we could not confirm whether the patients completely met the diagnostic criteria of NTM infection and underestimate the actual incidence of NTM disease. Thus, our data should be interpreted with caution. Fifth, this study did not compare the individual risk for NTM or MTB diseases between various types of TNFI. Differences in risk could be caused either by different types of TNFI, differences in patient characteristics, or use of other immunosuppressive agents. The NHIS database is limited because physician case reports are voluntary and no data on exposure to other drugs are collected. For these reasons, we did not attempt to calculate or compare rates of NTM disease among different TNFI.

Nonetheless, our study deserves to be highlighted as the first population-based, longitudinal cohort study in an MTB endemic area using propensity score matching to assess the association of NTM disease with TNFI usage in RA patients. With more than 6 years of follow-up, our analysis showed that the cumulative incidence of NTM was higher in TNFI-treated RA than in untreated RA. We have demonstrated that TNFI treatment is associated with the risk of developing NTM in RA patients in the MTB endemic area. Unlike MTB infection, there are no formal recommendations for screening and prevention for NTM infection prior to starting TNFI in RA patients. Thus, this study can serve to increase awareness of the necessity for early detection of NTM disease in TNFI-treated RA even in younger patients and those without high CCI scores.

## Methods

### Data source and study population

This study is a retrospective population-based cohort study which retrieved data from the NHIS, provided by the Korean government. The NHIS, as an obligatory health insurance system in South Korea, provides mandatory healthcare for the vast majority of the Korean population and collects healthcare database information on patient demographics, inpatient and outpatient usage, prescription records, and deaths^37,38^. For certain rare and chronic diseases, such as RA and MTB, the NHIS operates the RID registration program and offers financial support (up to 90% copayment) to patients who met the diagnostic criteria after the RID program registration. To qualify for enrollment in the RID program, patients had to meet the diagnostic criteria provided by the NHIS for each RID, and assessments had to be approved by specialized physicians. The diagnostic codes were defined according to the International Classification of Diseases, 10th revision (ICD-10), and a special code (V code) was assigned in the RID database^39^. Since the NHIS could refuse to pay hospital costs if diagnosis did not meet specific criteria, cases are reviewed by medical institutions prior to submission to the NHIS and a reliable diagnosis can be presumed. We identified RA patients using special code (V code) in the RID database, as this method has been shown to have higher accuracy than using only the ICD code^40,41^. There have also been several prior studies on the incidence and prevalence of other rare incurable disease using the same RID registration database^42–44^. Registration of RA in the RID database requires both a positive rheumatoid factor test and an official doctor’s report documenting that the patient fulfills the classification criteria of RA.

### Study design and definitions

From January 1, 2007 to December 31, 2016 we retrieved data from the NHIS database. We selected seropositive RA patients > 18 years of age fulfilling both the RA diagnosis code from the ICD10 (M05) and the V223 code in the RID database between July 1, 2009 and December 31, 2010 for inclusion in this study. The TNFI-treated RA group contained RA patients who had received at least one treatment with TNFIs, including infliximab, etanercept, or adalimumab and the untreated RA group contained patients not treated with any TNFIs during the study period.

We decided to establish a washout period to excluded cases previously treated with TNFIs or diagnosed with NTM and MTB. Previous studies reported that the risk of infection requiring hospitalization associated with TNFI was not statistically significant after two years of TNFI administration^45^, and that most cases of NTM and MTB occurred within three years of TNFI treatment^8,46,47^. Thus, the first 2.5 years from January 1, 2007 to June 30, 2009 were considered as a washout period. We excluded patients who had other rheumatic diseases including systemic lupus erythematosus (M32), Dermatomyositis, polymyositis (M33), Systemic sclerosis (M34), Sjogren's syndrome (M350), Ankylosing spondylitis (M45), Wegener's granulomatosis (M313), Churg-Strauss syndrome (M301), Polyarteritis nodosa (M300), Behcet's disease (M352), Kawasaki diseases (M303), Adult-onset Still's disease (M061, M082), Sarcoidosis (D86), Mixed connective tissue disease (M351), and those with other immunocompromised condition including malignancy (C00-C97), HIV (B20-B24) during the washout period. We also excluded a further 1946 patients who were treated with TNFI during the follow-up period, giving a total of 27,844 enrolled RA patients (Fig. 1).

We followed any newly diagnosed mycobacterial infection (including NTM and MTB) until December 31, 2016 (Supplementary Figure S1). MTB infection was detected based on its ICD-10 codes (A15-A19) and the MTB RID registration code, while NTM infection was defined as at least one principal diagnosis of NTM (ICD-10 code: A31).

The index date was defined as the date of the first prescription of TNFI in the TNFI-treated RA group and the first detection date of RA diagnosis in the untreated RA group during period of inclusion between July 1, 2009 and December 31, 2010. There were no cases previously treated with TNFI or diagnosed with NTM and MTB between July 1, 2009, and the index date in either the TNFI-treated RA group or untreated RA group. The follow-up period was from the index date to the date of detection of new mycobacterial infection or December 31, 2016, whichever was sooner.

Baseline patient demographic data obtained from the NHIS claims database were: age at index date, gender, and diagnosis of comorbidities using ICD-10 codes during the washout period. We calculated the Charlson comorbidity index (CCI), which encompasses 22 comorbid conditions, including cardiovascular disease, malignancy^48^. The scores for connective tissue disease were omitted from the CCI score in this study.

The study protocol was approved by the institutional review board (IRB) of Hanyang University Hospital, Seoul, South Korea (IRB No. 2018-12-015) according to the ethical guidelines of the Declaration of Helsinki. The requirement for informed consent from the participants was waived because the NHIS database was constructed after anonymization. All data used in this study were approved and provided in a blinded form by the NHIS (NHIS-2019-1-201).

### Statistical analysis

Data analysis compared distributions of demographic variables and the CCI score between the TNFI-treated and untreated RA groups. Descriptive statistics included means ± standard deviations for continuous variables and frequencies and percentages for categorical variables. Results were compared using t-test or Mann–Whitney U-test and the χ^2^ test or Fisher’s exact test, as appropriate. Incidence rates of NTM and MTB were calculated for both groups (per 100,000 PY), and incidence rates of NTM were stratified by demographic variables and CCI scores.

Controls for each TNFI-treated RA patient (1:4 pairs) were identified through propensity score matching based on gender, age, and CCI scores. Propensity score matching was performed by Greedy matching with using a caliper of 0.2 standard deviations of the logit of the propensity score. To assess the effect of TNFI on incidence of mycobacterial infection, the follow-up PY was calculated for each patient. We used the Poisson regression model to test the different incidence density of outcome (NTM or MTB) between TNFI-treated and untreated RA groups and presented the result as the incidence rate ratio (IRR) and 95% confidence interval (95% CI) between groups. We also used multivariable Cox proportional hazards regression analysis to measure the adjusted hazard ratio (aHR) of NTM and MTB with 95% CI for TNFI-treated RA compared to untreated RA, while controlling for age, gender, and CCI score. The Kaplan–Meier method was used to plot the cumulative proportions of NTM cases during the follow-up period, and the log-rank test was used to assess the differences between both groups. All analyses were conducted using SAS version 9.4 (SAS Institute, Cary, NC, USA). All tests were two-sided and *p*-values < 0.05 were considered statistically significant.

## Supplementary Information

Supplementary Information 1.

Supplementary Information 2.
