# Supplementary material for: TNF inhibitors increase the risk of nontuberculous mycobacteria in patients with seropositive rheumatoid arthritis in a mycobacterium tuberculosis endemic area
Source: Sci Rep. 2022 Mar 7;12:4003. doi: 10.1038/s41598-022-07968-w (PMC8901670; doi:10.1038/s41598-022-07968-w)
Supplement: Supplementary file 1 — Supplementary Information 1. [file 41598_2022_7968_MOESM1_ESM.doc]

**Supplement figure legends**

**Supplement figure S1.** Study design schema

We enrolled TNFI-treated RA patients and untreated RA patients between July 1, 2009 and December 31, 2010. We next established a washout period from January 1, 2007 to June 30, 2009 by excluding cases that had received TNFI treatment or were previously diagnosed with MTB or NTM. The index date was defined as the first day of RA diagnosis in the TNFI-untreated RA group (1) and as the date of the first prescription of TNFI in the TNFI-treated RA group (2). The follow-up period was from the index date to the date of detection of new mycobacterial infection or December 31, 2016, whichever was sooner. We further excluded patients who were not treated with TNFI at enrollment but newly started TNFI during the follow-up period (dashed arrow). MTB, mycobacterium tuberculosis; NTM, nontuberculous mycobacterium; TNFI, tumor necrosis factor inhibitor.
